# Simple model for estimation of absorbed dose by organs and tumors after PRRT from a single SPECT/CT study

**DOI:** 10.1186/s40658-021-00409-z

**Published:** 2021-08-26

**Authors:** Alexandre Chicheportiche, Moshe Sason, Jeremy Godefroy, Yodphat Krausz, Mahmoud Zidan, Kira Oleinikov, Amichay Meirovitz, David J. Gross, Simona Grozinsky-Glasberg, Simona Ben-Haim

**Affiliations:** 1 Department of Nuclear Medicine & Biophysics, Hadassah Medical Organization and Faculty of Medicine, Hebrew University of Jerusalem, 91120 Jerusalem, Israel; 2 Neuroendocrine Tumor Unit, ENETS Center of Excellence, Endocrinology and Metabolism Department, Hadassah Medical Organization and Faculty of Medicine, Hebrew University of Jerusalem, 91120 Jerusalem, Israel; 3 Oncology Department and Radiation Therapy Unit, Hadassah Medical Organization and Faculty of Medicine, Hebrew University of Jerusalem, 91120 Jerusalem, Israel; 4 Institute of Nuclear Medicine, University College London Hospitals, London, UK

## Abstract

**Background:**

Following each cycle of peptide receptor radionuclide therapy (PRRT), absorbed doses by tumors and normal organs are typically calculated from three quantitative single-photon emission computed tomography (SPECT)/computed tomography (CT) studies acquired at *t*_1_ = 24 h, *t*_2_ = 96 h, *t*_3_ = 168 h after the first cycle of treatment and from a single study at *t*_1_ after the subsequent cycles. In the present study, we have assessed the feasibility of a single SPECT/CT study after each PRRT cycle using a trained multiple linear regression (MLR) model for absorbed dose calculation and have evaluated its impact on patient management. Quantitative [^177^Lu]-DOTA-TATE SPECT/CT data after PRRT of seventy-two consecutive metastatic neuroendocrine tumors patients were retrospectively evaluated. A set of 40 consecutive studies was used to train the MLR model. The two independent variables of the model included the time of imaging after administration of the treatment and the radiopharmaceutical activity concentration in a given  organ/tumor. The dependent variable was the dose absorbed by the organ/tumor obtained with the standard protocol. For bone marrow dosimetry, the independent variables included the time of imaging, and the blood and remainder of the body activity concentration. The model was evaluated in 32 consecutive patients. Absorbed doses were assessed for kidneys, bone marrow, liver, spleen and tumor sites.

**Results:**

There was no difference in management decisions, whether PRRT can be safely continued or not because unsafe absorbed dose to risk organs between the standard and the MLR model-based protocol using a single SPECT/CT study performed at *t*_3_ = 168 h after the first cycle and at *t*_1_ = 24 h after the subsequent cycles. Cumulative absorbed doses were obtained with mean relative differences of − 0.5% ± 5.4%, 1.6% ± 15.1%, − 6.2% ± 7.3%, − 5.5% ± 5.8% and 2.9% ± 12.7% for kidneys, bone marrow, liver, spleen and tumors, respectively (Pearson’s *r* correlation coefficient 0.99, 0.91, 0.99, 0.99 and 0.97, respectively).

**Conclusion:**

Dosimetry calculations using a MLR model with a single SPECT/CT study are in good agreement with the standard protocol, while avoiding the use of dosimetry software and enabling improved patient comfort and reduced scanner and staff time.

**Supplementary Information:**

The online version contains supplementary material available at 10.1186/s40658-021-00409-z.

## Background

The radionuclide ^177^Lu has been proved useful in peptide-targeted receptor radionuclide therapy (PRRT) because of its favorable decay characteristics and the possibility of reliable labeling of biomolecules used for tumor targeting. ^177^Lu decays to ^177^Hf (hafnium) with a half-life of 6.65 days by *β*^−^ decay [1]. The maximum kinetic energy of the emitted *β*^−^ particles is 497 keV with a mean kinetic energy of approximately 134 keV [2]. Thanks to the energy deposited by the *β* particles in tumors, [^177^Lu]-DOTA-TATE therapy has been shown to be have an effective therapeutic effect in metastatic NETs [3–5]. Additionally to *β*^−^ emissions, ^177^Lu emits γ-photons (main energy of 208.4 keV (10.36%)) allowing for post-treatment imaging and personalized dosimetry calculation. [^177^Lu]-DOTA-TATE therapy is typically given as a treatment of four fixed cycles of 7.4 GBq (200 mCi), 6–12 weeks apart between each injection [3, 6–8]. The aim is to obtain an optimal therapeutic effect to tumors and a minimum absorbed dose to the healthy organs at risk, mainly kidneys and bone marrow. However, the maximum safety absorbed dose threshold for kidneys is not well defined and some authors define it at 23 Gy [9, 10], while others argue for 30 Gy [11, 12]. For bone marrow, the maximum absorbed dose threshold is defined at 2 Gy although it is rarely a limiting factor [9, 13]. In our institution, dosimetry calculations are performed after each PRRT cycle in order to assess the cumulative absorbed dose to organs at risk and tumors, and to guide management decisions. The PRRT treatment series is stopped if the cumulative dose absorbed by kidneys and bone marrow is expected to exceed 25 Gy and 2 Gy, respectively [13, 14].

Quantitative single-photon emission computed tomography (SPECT) images corrected for photon attenuation (from CT attenuation maps), scattered photons and blurring (resolution recovery) enable calculation of the absorbed doses by the patient’s organs and tumors [15–17]. According to EANM/MIRD guidelines [15], three SPECT/CT studies are acquired after the first cycle at *t*_1_ = 24 h, *t*_2_ = 96 h and *t*_3_ = 168 h after the radiopharmaceutical injection and a single SPECT/CT study at *t*_1_ = 24 h after the subsequent cycles, assuming an unchanged effective half-life of [^177^Lu]-DOTA-TATE [18, 19]. Radioactivity concentration from blood samples after each cycle of treatment is used to estimate bone marrow absorbed dose [20].

Different methods were suggested to reduce the number of patient visits and post-treatment studies after PRRT. A single imaging time point from the second treatment cycle has been proposed, assuming no change in the effective half-life of [^177^Lu]-DOTA-TATE [18, 19]. In a recent work [14], we assessed the feasibility of a two-time point dosimetry protocol with SPECT/CT studies acquired at *t*_1_ and *t*_3_ after the first cycle of treatment and at *t*_1_ after the following ones. Willowson et al. [19] suggested to calculate renal dosimetry using a single SPECT/CT study and a mean renal clearance half-time. This method led to large mean deviations of absorbed dose estimates (minimum 30% error), but another study [21] showed that the use of a single SPECT/CT acquired 96 h after injection was reliable to estimate absorbed doses. In the present study, the feasibility of a “one-time point” protocol using a multiple linear regression (MLR) model for calculation of the doses absorbed by kidneys, bone marrow, liver, spleen and tumors was assessed. In addition, we have evaluated the impact of the single time point protocol for dosimetry calculation on patient management.

## Methods

### Patients

In our institution, between May 2018 and November 2020, 262 [^177^Lu]-DOTA-TATE therapy cycles were administered to 93 consecutive metastatic neuroendocrine tumors patients. The data were divided into a training dataset, used for the development of the model, and a test set, used to evaluate the model.

Inclusion criteria for the training dataset were: (a) patients who started and completed their series of PRRT cycles during this time period and (b) who underwent at least one PRRT cycle with post-treatment imaging at (*t*_1_, *t*_3_) or (*t*_1_, *t*_2,_ *t*_3_) for dosimetry calculations. Eighty-two of the 93 patients completed their therapy during this period in our center. Two patients were hospitalized and one  died before completion of post-treatment scans after the first cycle of treatment. One additional patient was excluded due to missing data on hospital archiving system. Of the 78 remaining patients, the data of the first cycle (including multiple time points) of therapy of the first 40 consecutive patients (22 men, 18 women; average age: 63 years, range 16–86 years) were used to train the model.

**Fig. 1:**
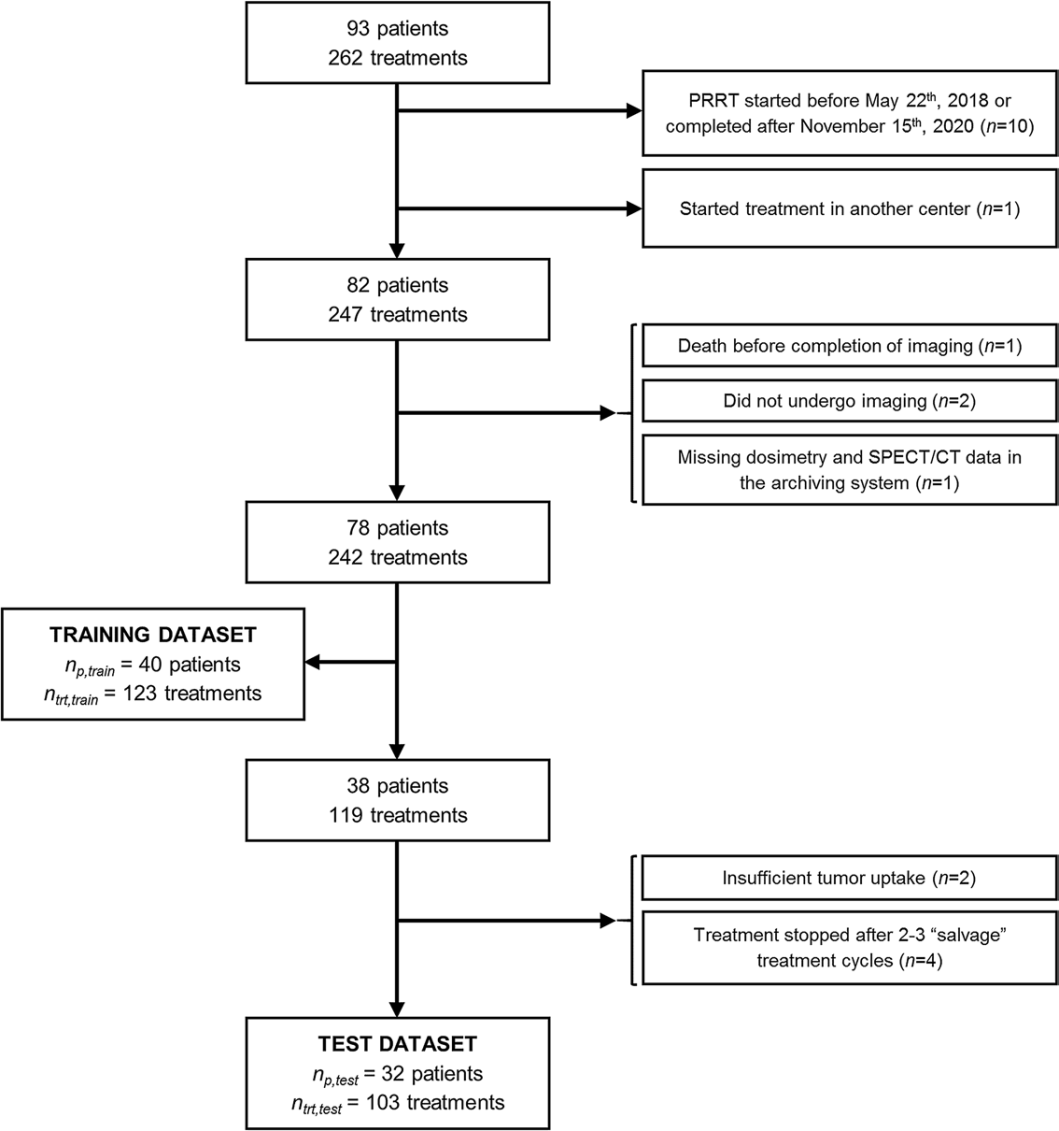
Chart of patient inclusion. *n*_p,train_ and *n*_p,test_ represent the number of patients included in the training and test dataset, respectively. *n*_trt,test_ represents the number of treatment cycles included in the test dataset

Inclusion criteria for the test dataset were: (a) patients who started and completed their series of treatments during the same period and (b) patients for whom the sole reason of treatment discontinuation was treatment toxicity or an expected absorbed dose higher than 25 Gy to kidneys or than 2 Gy to bone marrow. Of the 38 remaining patients, two patients with insufficient radiopharmaceutical uptake in tumors and four patients who had received previous PRRT and were referred for “salvage” treatment, regardless of the kidney absorbed dose, were excluded. The remaining 32 patients (18 men, 14 women; average age: 63 years, range 18–79 years) included in the test dataset have received a total of 103 therapy cycles (four cycles in 17, three cycles in nine, two cycles in two and one cycle in four patients). In nine of 15 patients who did not complete 4 cycles of PRRT (60%), therapy was stopped because of high kidney absorbed dose. In the remaining 6/15 (40%) patients, therapy was stopped because of general deterioration. Clinical characteristics of the 72 patients (Fig. 1) are summarized in Table 1.

**Table 1:** Demographic data for the patients included in the study

Characteristic	Total	Training dataset	Test dataset
Total number of patients	72	40	32
*Age (years)*			
Mean ± standard deviation	63 ± 13	63 ± 12	63 ± 14
Range	16–86	16–86	18–79
*Gender*			
Male	40	22	18
Female	32	18	14
*Primary tumor site*			
Pancreas	25	13	12
Small bowel	16	7	9
Lung	8	5	3
Pheochromocytoma	5	3	2
Unknown	6	3	3
Thymus	3	2	1
Stomach	2	2	0
Rectum	2	2	0
Carotid paraganglioma	2	1	1
Appendix	1	0	1
Colon	1	1	0
Esthesioneuroblastoma	1	1	0
*Sites of metastases*			
Liver	53	28	25
Lymph nodes	36	18	18
Bones	35	25	10
Lung	9	7	2
Peritoneum	6	3	3
Ovaries	2	0	2
Thyroid	1	1	0
Adrenal	1	1	0
Pelvic mass	1	0	1
Spleen	1	0	1
Pancreas	1	0	1

### PRRT therapy

[^177^Lu]-DOTA-Octreotate was locally prepared (S.R.Y Ltd, Jerusalem, Israel). Infusion of 1.5 L of amino acids solution (Arginine/lysine, 6.25 Gms, Concept for Pharmacy Ltd.) at a rate of 200 mL/hr started at least thirty minutes prior to [^177^Lu]-DOTA-TATE administration. The radioactive ligand was co-administered intravenously over 30 min. The mean activity per cycle of treatment was 7.3 ± 0.5 GBq (198.3 ± 14.3 mCi) with a median cumulative activity per patient of 23.4 GBq (6.8–45.9 GBq). The interval between treatment cycles was 5–13 weeks (median = 7 weeks).

### Post-treatment imaging

Post-treatment imaging was performed as described in previous works [14, 22]. Briefly, after each PRRT cycle, a planar whole body and quantitative SPECT/CT of the abdomen including kidneys, liver and spleen were acquired. All images were acquired on a Discovery NM/CT 670 scanner (General Electric Medical Systems, Haifa, Israel) with Medium Energy General Purpose collimators with a 20% energy window around the main photopeak of ^177^Lu (208 keV) [23]. Scatter correction was performed using a second energy window placed ± 10% around 166.4 keV. Whole body images were acquired in step-and-shoot mode (180 s per view) in a 256 × 1024 matrix. SPECT imaging was performed with 60 views over 360°, 30 s/frame (15 min acquisition/FOV) in a 128 × 128 matrix. CT was acquired before each SPECT acquisition with the integrated BrightSpeed multidetector CT) using a tube voltage of 120 kV and the Smart current option (80–220 mA—Noise Index: 17). SPECT images were calibrated as previously described [13]. Briefly, calibration of SPECT images was based on a series of SPECT acquisitions of a 20-mL ^177^Lu vial placed in the center of the gamma camera FOV with an activity ranging from about 110 MBq (2.7 mCi) to 6660 MBq (180 mCi). The ^177^Lu calibration source was placed in the center of 8 1-L saline bags with two additional ^177^Lu sources in order to simulate an amount of scatter similar to a clinical scan. However, recovery coefficients were not determined with this method and no correction for partial volume effect (PVE) was performed.

### Bone marrow dosimetry

Bone marrow dosimetry was calculated as previously described [22]. Blood samples were drawn at *t*_1_ and *t*_3_ after the first cycle and at *t*_1_ following subsequent cycles. The blood activity concentration was fitted by a mono-exponential curve to estimate the self-dose to the bone marrow.

### Image analysis and standard dosimetry calculation

Image analysis for dosimetry was performed as previously described [13, 14, 22] using the General Electric Dosimetry Toolkit (GE DTK) software on Xeleris 3.0 Workstation (General Electric Medical Systems, Haifa, Israel). Images were reconstructed with Ordered Subsets Expectation Maximization algorithm (2 iterations, 10 subsets), attenuation correction (from CT), scatter correction and resolution recovery (for blurring). Organs and tumors were delineated on functional (SPECT) or anatomical (CT) images. Volumes of interest (VOIs) were placed over the entire healthy organs area of interest (kidneys, liver, spleen and remainder of the body) and over tumors.

Absorbed doses were computed using an in-house code, taking as input the volume and activity concentrations in each VOI at every time point given by GE DTK. The code performs mono-exponential curves fitting from multiple time points [18, 19] and calculates residence times in the different organs and tumors.

In the standard dosimetry protocol, dose absorbed by the tumors was computed as previously described [16]. For healthy organs (kidneys, liver, spleen, bone marrow), the absorbed doses were computed using the medical internal radiation dose (MIRD) formalism [24] as follows:

1 $$D\left({r}_{k}\right)= {\tilde{A }}_{k}\cdot DF\left({r}_{k}\leftarrow {r}_{k}\right)+ \sum_{s\ne k}{\tilde{A }}_{s}\cdot DF\left({r}_{k}\leftarrow {r}_{s}\right)$$

where $D\left({r}_{k}\right)$ is the dose absorbed in the target organ *r*_*k*_ in [mGy]; ${\tilde{A }}_{k}$ and ${\tilde{A }}_{s},$ respectively, are the cumulated activities in the target *r*_*k*_ and source *r*_*s*_ organs in [MBq s]; and DF(*b* ← *a*) is the dose factor for a couple source *a*–target *b* in [mGy]/[MBq s]. The dose factors were taken from OLINDA/EXM 1.0 [25] for the adult male and adult female phantoms [22].

### Multiple linear regression models

#### Solid organs and tumors

Absorbed dose to solid organs (kidneys, liver, spleen) and tumors is essentially due to self-dose, with a negligible contribution of the cross-dose [16, 26] and therefore corresponds mainly to the first term of Eq. (1). Moreover, expressing the time-dependent activity *A*_*k*_(*t*) in the target *r*_*k*_ as a mono-exponential function, the cumulative activity ${\tilde{A }}_{k}$ simplifies to ${A}_{k}(t=0)/{\lambda }_{k}$, where ${\lambda }_{k}$ is the effective decay constant of [^177^Lu]-DOTA-TATE in the target. The dose factor $\mathrm{DF}\left({r}_{k}\leftarrow {r}_{k}\right)$ can be expressed in function of the mass ${m}_{k}$ of the target organ [kg] and physical constants, namely the equilibrium dose constant ${\Delta }_{i}$ in [kg mGy/MBq s] for particles of a particular type and energy, here indicated by *i* and the absorbed fraction ${\varnothing }_{i}\left({r}_{k}\leftarrow {r}_{k}\right)$ as [24]:

2 $$\mathrm{DF}\left({r}_{k}\leftarrow {r}_{k}\right)=\frac{1}{{m}_{k}}\sum_{i}{\Delta }_{i}{\varnothing }_{i}\left({r}_{k}\leftarrow {r}_{k}\right)= \frac{1}{{m}_{k}}\cdot {\Theta }_{k,k}$$

with ${\Theta }_{k,k}=\sum_{i}{\Delta }_{i}{\varnothing }_{i}\left({r}_{k}\leftarrow {r}_{k}\right)$. The dose absorbed by the target *r*_*k*_ can therefore be written as:

3 $$D\left({r}_{k}\right)=\frac{{A}_{k}(t=0)}{{m}_{k} \cdot {\lambda }_{k}} \cdot {\Theta }_{k,k}=\frac{{A}_{k}({t}_{s}) \cdot {e}^{{\lambda }_{k}\times {t}_{s}}, }{{m}_{k} \cdot {\lambda }_{k}}\cdot {\Theta }_{k,k}$$

and then estimated from a single quantitative SPECT/CT study performed at *t* = ${t}_{s}$ where the activity and the mass of the target ${m}_{k}$ are obtained from a VOI drawn around the organ or tumor. A MLR model with two independent variables; $\mathrm{ln}\left(\frac{{A}_{k}\left({t}_{s}\right)}{{m}_{k}}\right)\approx \mathrm{ln}\left(\frac{{S\cdot C}_{k}\left({t}_{s}\right)}{{V}_{k}}\right)$ (*S* is the SPECT sensitivity in [MBq/cps] and ${C}_{k}({t}_{s})$ and ${V}_{k}$ are the measured counts per second [cps] and volume [cc] in the target VOI, respectively) and ${t}_{s}$, and one dependent variable; $\mathrm{ln}\left(D\left({r}_{k}\right)\right)$—can be written:

4 $$\mathrm{ln}\left(D\left({r}_{k}\right)\right)\sim {\alpha }_{0,k}+{\alpha }_{1,k}\mathrm{ln}\left(\frac{{S\cdot C}_{k}\left({t}_{s}\right)}{{V}_{k}}\right)+{\alpha }_{2,k} {t}_{s}$$

where ${\alpha }_{0,k}$, ${\alpha }_{1,k}$ and ${\alpha }_{2,k}$ are the regression coefficients.

#### Bone marrow

The largest contribution to the absorbed dose in the bone marrow is derived from the self-dose conveyed by the blood, followed by the cross-dose from the remainder of the body [9]. Therefore, the dose absorbed by the bone marrow $D\left(\mathrm{BM}\right)$ can be formulated as:

5 $$D\left(\mathrm{BM}\right)= \frac{{a}_{\mathrm{blood}}({t}_{s}) \cdot {e}^{{\lambda }_{\mathrm{blood}}\times {t}_{s}}, }{ {\lambda }_{\mathrm{blood}}}\cdot {\Theta }_{\mathrm{BM,BM}}+\frac{{A}_{\mathrm{RM}}({t}_{s}) \cdot {e}^{{\lambda }_{\mathrm{RM}}\times {t}_{s}}, }{{m}_{\mathrm{RM}} \cdot {\lambda }_{\mathrm{RM}}}\cdot {\Theta }_{\mathrm{BM,RM}}$$

where ${a}_{\mathrm{blood}}({t}_{s})$ is the blood activity concentration in [MBq/cc], ${A}_{\mathrm{RM}}({t}_{s})$ the activity at time ${t}_{s}$ in the remainder of the body in [MBq] or [mCi] and ${m}_{\mathrm{RM}}$ the mass of the remainder of the body. ${\lambda }_{\mathrm{blood}}$ and ${\lambda }_{\mathrm{RM}}$ are the effective decay constants of the radiopharmaceutical in the blood and remainder of the body, respectively. After estimating ${\Theta }_{\mathrm{BM,BM}}$ and ${\Theta }_{\mathrm{BM,RM}}$ from the activities, masses and decay constants data obtained with our standard dosimetry calculation method, we hypothesize that the absorbed dose $D\left(\mathrm{BM}\right)$ can be approximated by:

6 $$D\left(\mathrm{BM}\right)\sim \left[{a}_{\mathrm{blood}}\left({t}_{s}\right)\cdot {\Theta }_{\mathrm{BM,BM}}+\frac{S\cdot {C}_{\mathrm{RM}}({t}_{s})}{{V}_{\mathrm{RM}} }\cdot {\Theta }_{\mathrm{BM,RM}} \right]\frac{{e}^{{\lambda }_{\mathrm{BM}}\times {t}_{s}} }{{\lambda }_{\mathrm{BM}}}$$

where ${\lambda }_{\mathrm{BM}}$ is a total effective decay constant for bone marrow, ${C}_{\mathrm{RM}}({t}_{s})$ is the measured cps in the remainder of the body VOI, and ${V}_{\mathrm{RM}}$ is volume of this VOI. A MLR model with two independent variables: $\mathrm{ln}\left[{a}_{\mathrm{blood}}\left({t}_{s}\right)\cdot {\Theta }_{\mathrm{BM,BM}}+\frac{S\cdot {C}_{\mathrm{RM}}({t}_{s})}{{V}_{\mathrm{RM}} }\cdot {\Theta }_{\mathrm{BM,RM}}\right]$ and ${t}_{s}$—and one dependent variable; $\mathrm{ln}\left(D\left({\mathrm{BM}}\right)\right)$—can be written as:

7 $$\mathrm{ln}\left(D\left({\mathrm{BM}}\right)\right)\sim {\beta }_{0,{\mathrm{BM}}}+{\beta }_{1,{\mathrm{BM}}}\mathrm{ln}\left(\left[{a}_{\mathrm{blood}}\left({t}_{s}\right)\cdot {\Theta }_{\mathrm{BM,BM}}+\frac{S\cdot {C}_{\mathrm{RM}}({t}_{s})}{{V}_{\mathrm{RM}} }\cdot {\Theta }_{\mathrm{BM,RM}} \right]\right)+{\beta }_{2,{\mathrm{BM}}} {t}_{s}$$

where ${\beta }_{0,{\mathrm{BM}}}$, ${\beta }_{1,{\mathrm{BM}}}$ and ${\beta }_{2,{\mathrm{BM}}}$ are the regression coefficients.

A more detailed description of the methodology is available in Additional file 1: Appendix A.

### Model training, performance evaluation and statistical methods

#### Model training

The MLR models in Eqs. (4) and (7) were trained using the SPECT/CT and blood activity concentration data of the 40 NET patients included in the training dataset. These included 116 SPECT/CT studies (40 studies at *t*_1_ = 19 ± 2 h, 36 studies at *t*_2_ = 97 ± 20 h and 40 studies at *t*_3_ = 163 ± 18 h) and 40 blood samples at *t*_1_ and *t*_3_. The fit was performed on 40 kidneys, 40 bone marrows, 40 livers, 37 spleens and 39 tumors.

#### Model performance evaluation

Evaluation of the final models was performed on the test dataset with 32 SPECT/CT studies at *t*_1_ = 19 ± 2 h, 28 studies at *t*_2_ = 88 ± 34 h and 32 at *t*_3_ = 157 ± 30 h and 31 blood samples at *t*_1_ and *t*_3_ after the first cycle of treatment. A single SPECT/CT and blood sample at *t*_1_ = 19 ± 2 h were available for subsequent ycles. The predicted absorbed doses by the organs, tumors and bone marrow were calculated using a single SPECT/CT study at *t*_*s*_ and Eqs. (8) and (9), respectively, with the regression coefficients obtained during the model training.

8 $$D\left({r}_{k}\right) \sim {\left[\frac{{ S\cdot C}_{k}({t}_{study})}{{V}_{k}}\right]}^{{\alpha }_{1,k}}\cdot {e}^{{\alpha }_{2,k} {t}_{s} + {\alpha }_{0,k}}$$

9 $$D\left({\mathrm{BM}}\right) \sim {\left[{a}_{\mathrm{blood}}\left({t}_{s}\right)\cdot {\Theta }_{\mathrm{BM,BM}}+\frac{S\cdot {C}_{\mathrm{RM}}({t}_{s})}{{V}_{\mathrm{RM}} }\cdot {\Theta }_{\mathrm{BM,RM}} \right]}^{{\beta }_{1,{\mathrm{BM}}}}\cdot {e}^{{\beta }_{2,{\mathrm{BM}}} {t}_{s} + {\beta }_{0,{\mathrm{BM}}}}$$

The predicted absorbed doses after the first cycle of treatment were calculated for 32 kidneys, 31 bone marrows, 32 livers, 31 spleens and 32 tumors.

Patient management was evaluated with kidneys and bone marrow dosimetry calculated from a single SPECT/CT study at *t*_*s*_ = *t*_1_, *t*_2_ or *t*_3_ after the first cycle of treatment and at *t*_*s*_ = *t*_1_ after the following cycles to determine the best MLR model for dosimetry calculation. For consecutive therapy cycles, hypothetic activities in organs and tumors at *t*_*s*_ = *t*_2_ = 96 h and *t*_3_ = 168 h were calculated from the effective half-life obtained after the first cycle with the standard multiple time points calculation. When the cumulative absorbed doses to kidneys and bone marrow after the following cycle, estimated from the absorbed doses during previous treatments, are “expected” to exceed 25 Gy and 2 Gy for kidneys and bone marrow, respectively, no further PRRT is given, unless decided otherwise by a multi-disciplinary team. Patient management was also evaluated using the kidney absorbed dose “predicted” after the first or first two treatment cycles, allowing for an early decision regarding the number of cycles that can be administered safely [13].

#### Statistical analysis

Coefficients of determination ${r}_{\mathrm{train}}^{2}$ of the MLR fit model were calculated. To evaluate the MLR model, the coefficients of determination ${r}_{\mathrm{test}}^{2}$ between the absorbed doses calculated using the standard protocol and those predicted by the MLR model were calculated on the test dataset for the first cycle. Fitting of the MLR model and calculation of *r*^2^ were performed using RStudio statistical software, version 1.3.1056 and R version 4.0.2.

A one-sided binomial test was performed using the StatXact, Cytel Inc., Cambridge MA, version 10 20 software in order to test the power of the null hypothesis.

Differences between the absorbed doses by organs and tumors obtained using the standard and MLR-based protocols were assessed with Bland and Altman and correlation plots.

## Results

### Model training

The time of imaging and the activity concentrations included in the independent variables of Eqs. (4) and (7) were expressed in [h] and [mCi/cc], respectively. The dependent variable (absorbed dose) was expressed in [mGy]. Table 2 presents the regression coefficients ${\alpha }_{0,k}$, ${\alpha }_{1,k}$ and ${\alpha }_{2,k}$ and the corresponding ${r}_{\mathrm{train}}^{2}$ of Eq. (4) for kidneys, liver, spleen and tumors when using the SPECT/CT data at *t*_*f*_ = *t*_1_, *t*_2_ or *t*_3_ or combination of all time points for fitting *t*_*f*_ = (*t*_1_, *t*_2_, *t*_3_). Regression coefficients ${\Theta }_{\mathrm{BM,BM}}$, ${\Theta }_{\mathrm{BM,RM}}$, ${\beta }_{0,{\mathrm{BM}}}$, ${\beta }_{1,{\mathrm{BM}}}$ and ${\beta }_{2,{\mathrm{BM}}}$ and ${r}_{\mathrm{train}}^{2}$ using Eq. (7) for bone marrow are also presented when using independent data at *t*_*f*_ = *t*_1_ or *t*_3_ or both. These results show that the MLR model fit is improved when using a single SPECT/CT study at *t*_2_ for organs and tumor absorbed dose estimation. A single study performed at *t*_3_ gives similar results except for kidneys with ${r}_{\mathrm{train}}^{2}$= 0.95. For estimation of the dose absorbed by bone marrow, data at *t*_3_ lead to the best fit with ${r}_{\mathrm{train}}^{2}$= 0.92.

**Table 2:** Summary of the model training for kidneys, tumors, liver, spleen and bone marrow

	Model training									
	Solid organs and tumors									
	*n*_train_	${\alpha }_{0,k}$	${\alpha }_{1,k}$	${\alpha }_{2,k}$	${r}_{\mathrm{train}}^{2}$	*n*_train_	${\alpha }_{0,k}$	${\alpha }_{1,k}$	${\alpha }_{2,k}$	${r}_{\mathrm{train}}^{2}$
	*t*_*f*_ = *t*_1_					*t*_*f*_ = *t*_2_				
Kidneys	40	12.01	0.91	0.0153	0.85	36	11.87	0.85	0.0117	0.98
Tumors	39	13.27	1.03	− 0.0105	0.97	35	12.90	0.99	0.0070	0.99
Liver	40	12.37	0.96	0.0205	0.88	36	11.86	0.86	0.0111	0.97
Spleen	37	12.21	1.00	0.0336	0.97	33	12.48	0.93	0.0094	0.99
	*t*_*f*_ = *t*_3_					*t*_*f*_ (*t*_1_, *t*_2_, *t*_3_)				
Kidneys	40	11.83	0.83	0.0103	0.95	116	11.86	0.85	0.0111	0.94
Tumors	39	12.76	0.97	0.0081	0.99	113	12.80	0.99	0.0086	0.99
Liver	40	12.57	0.91	0.0077	0.97	116	12.26	0.90	0.0090	0.92
Spleen	37	12.52	0.94	0.0090	0.99	107	12.51	0.95	0.0094	0.98

*t*_*f*_	Bone marrow						
	*n*_train_	${\Theta }_{\mathrm{BM,BM}}$	${\Theta }_{\mathrm{BM,RM}}$	${\beta }_{0,{\mathrm{BM}}}$	${\beta }_{1,{\mathrm{BM}}}$	${\beta }_{2,{\mathrm{BM}}}$	${r}_{\mathrm{train}}^{2}$
*t*_1_	40	1868	319	4.26	1.07	0.0241	0.78
*t*_3_	40	1889	315	5.03	0.82	0.0069	0.92
(*t*_1_, *t*_3_)	80	1879	316	4.59	0.90	0.0101	0.83

*t*_*f*_ and *n*_train_ represent, respectively, the time points considered for model training and the number of studies included in each case. *α*, *β* and $\Theta$ represent the regression coefficients obtained for a given organ/tumor and *t*_*f*_. Corresponding coefficients of determination ${r}_{\mathrm{train}}^{2}$ are also shown

### Model performance evaluation

The predicted absorbed doses were calculated with the SPECT/CT data of the test dataset performed at *t*_*s*_ = *t*_1_, *t*_2_ or *t*_3_ separately. Coefficients of determination ${r}_{\mathrm{test}}^{2}$ between the first cycle absorbed doses calculated using the standard protocol and the MLR model using equations at *t*_*f*_ and a single SPECT/CT study at *t*_*s*_ are presented in Table 3. These results suggest that, after the first therapy cycle, absorbed dose predictions at late times give better agreements for liver, spleen, bone marrow and tumors. For kidneys, the best agreement is obtained for *t*_*s*_ = 96 h.

**Table 3:** Summary of the model evaluation for kidneys, tumors, liver spleen and bone marrow

	Model evaluation											
	Solid organs and tumors											
	*t*_*f*_ = *t*_1_ and *t*_*s*_ = *t*_1_		*t*_*f*_ = *t*_2_ and *t*_*s*_ = *t*_2_		*t*_*f*_ = *t*_3_ and *t*_*s*_ = *t*_3_		*t*_*f*_ = (*t*_1_, *t*_2_, *t*_3_) for model fitting					
							*t*_*s*_ = *t*_1_		*t*_*s*_ = *t*_2_		*t*_*s*_ = *t*_3_	
	*n*_test_	${r}_{\mathrm{test}}^{2}$	*n*_test_	${r}_{\mathrm{test}}^{2}$	*n*_test_	${r}_{\mathrm{test}}^{2}$	*n*_test_	${r}_{\mathrm{test}}^{2}$	*n*_test_	${r}_{\mathrm{test}}^{2}$	*n*_test_	${r}_{\mathrm{test}}^{2}$
Kidneys	32	0.85	28	0.96	32	0.95	32	0.84	28	0.95	32	0.93
Tumors	32	0.88	28	0.95	32	0.99	32	0.88	28	0.97	32	0.98
Liver	32	0.71	28	0.93	32	0.95	32	0.67	28	0.92	32	0.95
Spleen	31	0.92	27	0.98	31	0.99	31	0.93	27	0.95	31	0.99

	Bone marrow							
	*t*_*f*_ = *t*_1_ and *t*_*s*_ = *t*_1_		*t*_*f*_ = *t*_3_ and *t*_*s*_ = *t*_3_		*t*_*f*_ = (*t*_1_, *t*_3_) for model fitting			
					*t*_*s*_ = *t*_1_		*t*_*s*_ = *t*_3_	
	*n*_test_	${r}_{\mathrm{test}}^{2}$	*n*_test_	${r}_{\mathrm{test}}^{2}$	*n*_test_	${r}_{\mathrm{test}}^{2}$	*n*_test_	${r}_{\mathrm{test}}^{2}$
Bone marrow	31	0.91	31	0.97	31	0.87	31	0.96

*n*_test_ represents the number of data considered for evaluation of the model. The time points *t*_*f*_ and *t*_*s*_ represent, respectively, the time considered for model training and for calculation of the absorbed dose using the MLR equation at *t*_*f*_. The coefficients of determination ${r}_{\mathrm{test}}^{2}$ are also shown

**Fig. 2:**
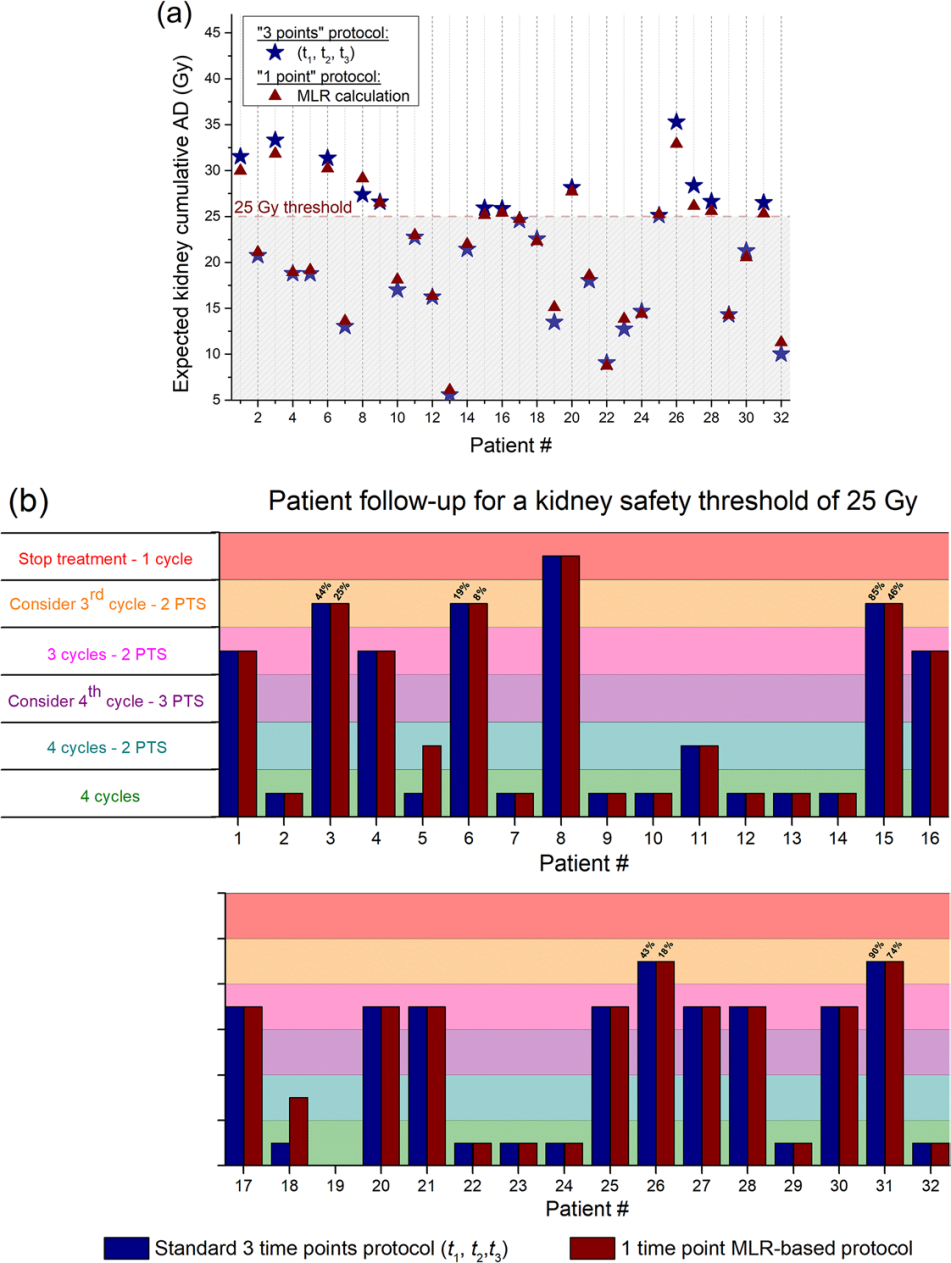
Patient management using the standard dosimetry protocol versus MLR-based protocol. **a** Expected kidney absorbed dose after the next PRRT cycle calculated from previous treatment cycles (1 to 3) using the standard (blue stars) or the MLR-based (triangles) protocols. **b** Management of the 25 patients included in the study based on the kidney cumulative “predicted” absorbed dose using either the standard or the MLR-based protocols with a safety threshold fixed at a 25 Gy. No management decision could be given for patient #19 with both protocols. Horizontal lines separate the different managements of the patient (4 CYCLES: the patient can receive 4 cycles safely, 1 PTS only; 4 CYCLE—2 PTSs: 4 cycles safely, 2 PTSs only; CONSIDER A fourth CYCLE—3 PTSs: consider to administer a fourth cycle based on the probability to exceed 25 Gy after 4 cycles; 3 CYCLES—2 PTSs: 3 cycles safely, 2 PTSs only; CONSIDER A third CYCLE—2 PTSs: consider at maximum a third cycle; STOP TREATMENT—1 cycle: stop after a single cycle). AD = absorbed dose

Patient management was evaluated using the equations obtained at *t*_*f*_ with a single SPECT/CT study at *t*_*s*_ = *t*_1_, *t*_2_ or *t*_3_ (*t*_1_ or *t*_3_ for bone marrow) after the first cycle of treatment and at *t*_*s*_ = *t*_1_ after the subsequent cycles. Hypothetic patient management obtained with the MLR model for *t*_*s*_ = *t*_2_ and *t*_3_ (*t*_3_ only for bone marrow) for cycle 2–4 was also evaluated. A total of 36 fit combinations were assessed to compare patient management (Additional file 2: Table S1). There was no change in patient management using the MLR protocol with the regression coefficients obtained at *t*_*f*_ = (*t*_1_, *t*_2_, *t*_3_) and with a single SPECT/CT study at *t*_*s*_ = *t*_3_ after the first cycle and using *t*_*f*_ = *t*_1_ and *t*_*s*_ = *t*_1_ after the following cycles. Indeed, all patients who continued or discontinued therapy according to the standard protocol would have been managed similarly with this MLR-based protocol (Fig. 2a). There was also no change in the patient management for bone marrow. For other fit combinations, changes in patient management decisions ranged from 1/28 to 6/32 (Additional file 2: Table S1). Patient management was also evaluated using the “predicted” cumulative absorbed dose algorithm for the follow-up of kidney dosimetry with the standard protocol and with the MLR protocol (Fig. 2b) and was found to be similar with differences in the number of post-treatment scans. For example, for patients #5 and #18, the standard protocol predicted after one cycle and one post-treatment scan (PTS) safe administration of four treatment cycles, while the MLR-based protocol predicted the same after two cycles of treatment and two PTSs.

**Fig. 3:**
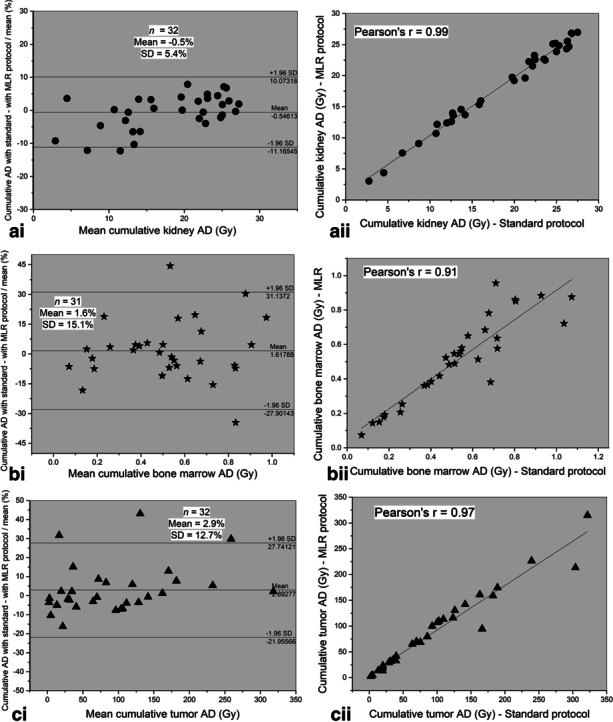
*(i)* Bland and Altman plots showing differences in the **a** kidney, **b** bone marrow and **c** tumor absorbed dose after completion of the treatment between the standard and MLR-based protocols, with 95% limits of agreement (mean ± 1.96 SD). Correlation plots are shown in (*ii*). AD = absorbed dose

Differences and correlation between the cumulative doses absorbed by kidneys (a), bone marrow (b) and tumors (c) using the MLR-based and the standard “3-time points” protocols are shown in Fig. 3. There is an excellent agreement between the two protocols for kidneys with a mean relative difference of − 0.3% ± 10.5% and − 0.5% ± 5.4% for the 103 kidney absorbed doses and the 32 cumulative kidney absorbed doses with a Pearson’s correlation coefficient *r* = 0.95 and *r* = 0.99, respectively (all *p* < 0.001). For bone marrow, a mean relative difference of 3.8% ± 17.8% and 1.6% ± 15.1% was obtained for the 100 bone marrows and 31 cumulative absorbed doses, with *r* = 0.90 and *r* = 0.91, respectively (*p* < 0.001). Similarly, a difference of 3.5% ± 19.3% (*r* = 0.96, *p* < 0.001) is obtained over the 103 tumors and of 2.9% ± 12.7% (*r* = 0.97, *p* < 0.001) over cumulative absorbed doses. The mean differences in the cumulative absorbed doses to liver and spleen were − 6.2% ± 7.3% and − 5.5% ± 5.8%, respectively, all with *r* = 0.99 (all *p* < 0.001) (Table 4).

**Table 4:** Mean relative errors in cumulative absorbed doses between the standard and MLR calculation for the kidneys, bone marrow, liver, spleen and tumors

1 point MLR versus Standard protocol	Kidneys	Bone marrow	Liver	Spleen	Tumors
Relative difference	− 0.5% ± 5.4%	1.6% ± 15.1%	− 6.2% ± 7.3%	− 5.5% ± 5.8%	2.9% ± 12.7%
Pearson’s *r*	0.99	0.91	0.99	0.99	0.97
Angular coefficient *a*	0.94	0.86	0.87	1.06	0.86

Pearson’s coefficients *r* and angular coefficients *a* are also shown

## Discussion

In this work, we aimed to improve the current dosimetry protocol by reducing the number of patient visits in the Nuclear Medicine clinic, thereby reducing scanner and staff time. The “1 time-point” MLR protocol based on regression coefficients calculated from *t*_*f*_ = (*t*_1_, *t*_2_, *t*_3_) and *t*_1_ and on data acquired at *t*_*s*_ = *t*_3_ and *t*_1_ for the first and following cycles, respectively, led to similar management decisions to the standard protocol. A sample size of 32 patients has a power of 96.6% to detect a true difference of at least 10% at the 0.05 level of significance, using a one-sided binomial test.

In our center, PRRT is administered in an inpatient isolation setting with patients staying overnight for radiation safety considerations and to perform the post-treatment study the next day. The best configuration should enable accurate dosimetry calculations from the studies performed the day after treatment, so patients would not need to return to the clinic for further imaging. However, we showed that patient management remained unchanged only when SPECT/CT was performed about one week after the first cycle and the day after PRRT for each subsequent cycle. Radiation safety considerations enable an outpatient setting for patients treated with 7.4 GBq (200 mCi) of [^177^Lu]-DOTA-TATE, attaining the radiation exposure release limit by 6 h after radiopharmaceutical administration [27, 28]. Therefore, with the MLR-based protocol, patients can be discharged within few hours after the first PRRT cycle, improving comfort, hospitalization-related expenses and staff exposure compared to the standard protocol. For each subsequent cycle, the MLR-based and the standard protocol is similar. The MLR-based protocol obviates the need of a dosimetry software for routine dosimetry calculations and reduces the calculation time. Indeed, a simple software allowing to draw VOIs and to obtain counts and volumes allows to calculate the absorbed doses by organs and tumors. Of course, calibration of the gamma camera is required for the calculation.

The equation used for bone marrow dosimetry calculation (Eq. 9) requires blood activity concentration and remainder of the body data. The estimation of the dose absorbed by bone marrow was performed by taking as input the blood activity concentration data only and considering the blood self-dose as the main contributor. Low coefficients of determination (average of 0.59) were obtained compared to the training set.

From Eqs. (3) and (5), the regression coefficient ${\alpha }_{2,k}$ corresponds to the effective decay constant ${\lambda }_{k}$ for organ or tumor ${r}_{k}$. Comparing the regression coefficients ${\alpha }_{2,k}$ (Table 2) to the effective decay constants λ_*k*_, calculated with the standard protocol, same order of magnitude was obtained. Indeed, in our population of 72 patients the mean effective decay constants of 0.0129 h^−1^, 0.0098 h^−1^,

0.0095 h^−1^ and 0.0082 h^−1^ were obtained with the standard “3 time points” protocol compared to regression coefficients of 0.0110 h^−1^, 0.0090 h^−1^, 0.0094 h^−1^ and 0.0086 h^−1^ for kidneys, liver, spleen and tumors, respectively, with *t*_*f*_ = (*t*_1_, *t*_2_, *t*_3_).

Reducing the number of PTSs after PRRT has been assessed by others. Using a MLR model with 3 PTSs after cycle 1 and a single time point for the following cycles caused relative errors of 2 ± 16% in the kidney absorbed dose estimation [19]. In the present study with a single PTS, the error in kidney absorbed dose estimation was − 0.5% ± 5.4%. The same authors showed that use of an average kidney effective half-life and a single PTS leads to a minimum mean relative error of 30% in the absorbed dose estimation [19] much higher than the deviations in present study. Hänscheid et al*.* [21] showed that based on a theoretical approach, a single post-treatment SPECT/CT study performed 4 days after the injection leads to reliable integral time estimation (self-dose) with median errors of 5% (range − 9 to + 17%) for kidneys, 6% (range − 7 to + 12%) for livers, 8% (range + 2 to + 20%) for spleens and 6% (range − 11 to + 16%) for the lesions. Median errors of percentage deviation in cumulative absorbed dose estimation (model/standard – 1) obtained in the present study were lower for kidneys and tumors, − 0.1% (range − 8 to 12%) and 0.8% (range − 43 to 16%), respectively, and similar for livers and spleens, 8% (range − 16 to 19%) and 6% (range − 8 to 16%), respectively. Although the results obtained in this study for the first cycle (Table 3) are similar to those obtained by Hänscheid et al*.* [21] with a best ${r}_{\mathrm{test}}^{2}$ for kidneys at *t*_*s*_ = 96 h and at *t*_*s*_ = 168 h for other organs and tumors, our conclusions are based on the comparison of the kidney cumulative absorbed dose follow-up. It seems that the best agreement between the MLR and the standard protocol (including a 3-time point study after the first cycle only and then single time point studies) is obtained as a combination of a late SPECT/CT acquisition at 168 h after the first cycle and an early SPECT/CT at 24 h after the subsequent cycles.

Present study has some limitations. Indeed, the validity of the regression coefficients of the MLR model is limited to the dosimetry protocol used in our institution (SPECT/CT studies acquired at 24 h, 96 h and 168 h after the first cycle). The MLR models proposed in this study should be tested and validated in new test dataset in other centers that would like to implement these models for dosimetry calculation. Also, the model should be trained on multiple time points data for all cycles and should be tested on single time point SPECT/CT studies in order to assess the accuracy of the methodology against a “full” dosimetry and to evaluate the impact on patient management. The best acquisition times may need to be redefined in this case. This would be especially important for patients with risk factors for kidney dysfunction who need to be monitored more carefully [18]. Furthermore, PRRT treatments with a maximum of 4 cycles of 7.4 GBq were considered in this study. However, even if in a near future more than 4 cycles are given and/or kidney toxicity threshold is increased, the low deviations dosimetry results of the MLR-based protocol from the standard one suggest that the new “one time-point” protocol could be used to predict the doses absorbed by organs and tumors with sufficient precision.

## Conclusion

Dosimetry calculations using a MLR model with a single SPECT/CT study acquired at 168 h after the first PRRT cycle and at 24 h after each additional PRRT are in good agreement with the standard imaging protocol. The MLR-based protocol did not lead to changes in patient management decisions, while it enables to improve patient comfort and to reduce treatment-related expenses, and scanner and staff time.

## Supplementary Information

**Additional file 1.** Detailed description of the Multiple Linear Regression (MLR) methodology for organ, tumor and bone-marrow absorbed doses calculation from a single SPECT/CT study.

**Additional file 2.** Number of cases the patient management would have been different between the MLR-based and standard dosimetry protocols for a given combination of (*t*_f_, *t*_s_) after the first treatment cycle and after the following ones.

## Data Availability

Patient imaging was done in the scope of the routine clinical diagnostic studies, and the raw data are stored in the hospital archiving system at the Hadassah-Hebrew University Medical Center, Jerusalem, Israel.

## Abbreviations

PRRT: Peptide receptor radionuclide therapy
SPECT: Single-photon emission computed tomography
CT: Computed tomography
MLR: Multiple linear regression
GE: General Electric
DTK: Dosimetry Toolkit
VOI: Volume of interest
MIRD: Medical internal radiation dose
BM: Bone marrow
PTS: Post-treatment scan
