# Supplementary material for: Simple model for estimation of absorbed dose by organs and tumors after PRRT from a single SPECT/CT study
Source: EJNMMI Phys. 2021 Aug 26;8:63. doi: 10.1186/s40658-021-00409-z (PMC8390741; doi:10.1186/s40658-021-00409-z)
Supplement: Supplementary file 1 — Additional file 1. Detailed description of the Multiple Linear Regression (MLR) methodology for organ, tumor and bone-marrow absorbed doses calculation from a single SPECT/CT study. [file 40658_2021_409_MOESM1_ESM.pdf]

Additional file 1: Appendix A. Detailed description of the Multiple Linear Regression (MLR) methodology for organ, tumor and bone-marrow absorbed doses calculation from a single SPECT/CT study.

With our standard dosimetry protocol, the medical internal radiation dose (MIRD) formalism is used to calculate the absorbed dose by healthy organs (kidneys, liver, spleen, bone marrow), as follows:

$$D(r_k) = \tilde{A}_k \cdot DF(r_k \leftarrow r_k) + \sum_{s \neq k} \tilde{A}_s \cdot DF(r_k \leftarrow r_s) \quad \text{(Eq. A1)}$$

with $D(r_k)$ the dose absorbed in the target organ r_k in [mGy]; $\tilde{A}_k$ and $\tilde{A}_s$ respectively the time integrated activity or cumulated activity in the target r_k and source organ r_s in [MBq·s], and DF(b←a) the dose factor for a couple source a-target b in [mGy]/[MBq·s]. The first and second terms of the equation correspond respectively to the self-dose and cross-dose contributions, i.e., the absorbed dose by the target organ r_k from disintegrations in the target itself and from disintegrations in other source organs, respectively.

Multiple Linear Regression model for organs and tumors

Considering that absorbed dose to solid organs (kidneys, liver, spleen) and tumors is essentially due to self-dose with a negligible contribution of the cross-dose, the MIRD formalism for organs and tumors r_k can be simplified to:

$$D(r_k) = \tilde{A}_k \cdot DF(r_k \leftarrow r_k) \quad \text{(Eq. A2)}$$

With the standard protocol, 3 SPECT/CT studies are acquired after the first therapy cycle. The time-dependent activity curve for organ r_k is modeled by a mono-exponential function fitting these points such that,

$$A_k(t) = A_k(0) \cdot e^{-\lambda_k \times t} \quad \text{(Eq. A3)}$$

with $A_k(0)$ the activity in organ r_k at time $t = 0$ and λ_k the effective decay constant of the radiopharmaceutical in the target. Therefore, the cumulative activity $\tilde{A}_k$ or integration of $A_k(t)$ over all time equals,

$$\tilde{A}_k = \int_0^{\infty} A_k(t)\, dt = A_k(0)/\lambda_k \qquad \text{(Eq. A4)}$$

According to equation A3, $A_k(0)$ can be written as,

$$A_k(0) = A_k(t) \cdot\ e^{+\lambda_k \times t} \qquad \text{(Eq. A5)}$$

And equation A4 becomes,

$$\tilde{A}_k = A_k(t)/\lambda_k \cdot\ e^{+\lambda_k \times t} \qquad \text{(Eq. A6)}$$

The dose factor $DF(r_k \leftarrow r_k)$ from equation A2 can be expressed as:

$$DF(r_k \leftarrow r_k) = \frac{1}{m_k}\sum_i \Delta_i \emptyset_i(r_k \leftarrow r_k) = \frac{1}{m_k} \cdot \Theta_{k,k} \qquad \text{(Eq. A7)}$$

where, m_k is the mass of the target organ [kg], Δ_i is the equilibrium dose constant for particles of a particular type and energy, here indicated by i [kg·mGy/MBq·s], $\emptyset_i(r_k \leftarrow r_k)$ represents the absorbed fraction of energy for a target organ r_k for particles i emitted from the same organ r_k and $\Theta_{k,k} = \sum_i \Delta_i \emptyset_i(r_k \leftarrow r_k)$. The dose absorbed by solid organs and tumors can therefore be estimated from equations A2, A6 and A7 as:

$$D(r_k) = \frac{A_k(t) \cdot e^{\lambda_k \times t},}{m_k \cdot \lambda_k} \cdot \Theta_{k,k} \qquad \text{(Eq. A8)}$$

The activity in the target $A_k(t)$ can be obtained from a quantitative SPECT/CT study performed at $t=t_s$ from a SPECT calibration factor (or sensitivity) S in [MBq/cps], as well as the mass of the target m_k can be estimated from the volume V_k in [cc] of a VOI drawn around the organ or tumor of interest (considering a tissue density of 1.0 g/cm^3). Equation A8 becomes:

$$D(r_k) \sim \frac{[S \cdot C_k(t_s)]}{V_k} \cdot e^{\lambda_k \times t_s} \cdot \frac{\Theta_{k,k}}{\lambda_k} \qquad \text{(Eq. A9)}$$

with, $C_k(t_s)$ the measured counts per second [cps] in the target organ or tumor VOI on the SPECT study at time t_s.

Applying natural logarithm of both sides of the equation A9, we obtain:

$$\ln\big(D(r_k)\big) \sim \ln\left(\frac{\Theta_{k,k}}{\lambda_k}\right) + \ln\left(\frac{S \cdot C_k(t_s)}{V_k}\right) + \lambda_k\, t_s \qquad \text{(Eq. A10)}$$

Therefore, a MLR model for organs and tumors with two independent known variables $\ln\left(\frac{S \cdot C_k(t_s)}{V_k}\right)$ and t_s and, one dependent variable $\ln(D(r_k))$ can be formulated as:

$$\ln(D(r_k)) \sim \alpha_{0,k} + \alpha_{1,k} \ln\left(\frac{S \cdot C_k(t_s)}{V_k}\right) + \alpha_{2,k}\, t_s \qquad \text{(Eq. A11)}$$

with $\alpha_{0,k}$, $\alpha_{1,k}$ and $\alpha_{2,k}$ the regression coefficients.

Multiple Linear Regression model for bone marrow

For bone marrow, the largest contribution is derived from the self-dose conveyed by the blood followed by cross-dose from the remainder of the body. The dose absorbed (equation A1) by the bone marrow $D(BM)$ can be written as:

$$D(BM) = \tilde{A}_{blood} \cdot DF(r_{BM} \leftarrow r_{BM}) + \tilde{A}_{RM} \cdot DF(r_{BM} \leftarrow r_{RM}) \qquad \text{(Eq. A12)}$$

$$\Leftrightarrow D(BM) \sim \frac{a_{blood}(t_s) \cdot e^{\lambda_{blood} \times t_s},}{\lambda_{blood}} \cdot \theta_{BM,BM} + \frac{A_{RM}(t_s) \cdot e^{\lambda_{RM} \times t_s},}{m_{RM} \cdot \lambda_{RM}} \cdot \theta_{BM,RM} \qquad \text{(Eq. A13)}$$

where $a_{blood}(t_s)$ is the blood activity concentration in [MBq/cc], $A_{RM}(t_s)$ the activity at time t_s in the remainder of the body in [MBq], m_{RM} the mass of the remainder of the body. λ_{blood} and λ_{RM} are the effective decay constants of the radiopharmaceutical for the blood and remainder of the body, respectively.

In equation A13, the unknown variables are $\theta_{BM,BM}$, $\theta_{BM,RM}$, λ_{blood} and λ_{RM}. Therefore, as a first step, from data (activities, masses and decay constants) obtained with our standard 3 time points dosimetry calculation method, a multiple linear regression has been performed in order to determine the coefficients $\Theta_{BM,BM}$ and $\Theta_{BM,RM}$, describing the relative contribution of the self and cross-doses to the bone marrow absorbed dose. In a second time, we hypothesize that the absorbed dose $D(BM)$ can be approximate as:

$$D(BM) \sim \left[a_{blood}(t_s) \cdot \theta_{BM,BM} + \frac{S \cdot C_{RM}(t_s)}{V_{RM}} \cdot \theta_{BM,RM}\right] \frac{e^{\lambda_{BM} \times t_s}}{\lambda_{BM}} \qquad \text{(Eq. A14)}$$

with λ_{BM} a total effective decay constant for bone marrow, $C_{RM}(t_s)$ the measured counts per second [cps] in the target remainder of the body VOI on the SPECT study at time t_s and V_{RM} the volume of this VOI [cc]. Similarly to equation A11, by applying the natural logarithm, a MLR model with two independent

variables $\ln\left[a_{blood}(t_s)\cdot\theta_{BM,BM}+\frac{S\cdot C_{RM}(t_s)}{V_{RM}}\cdot\theta_{BM,RM}\right]$ and t_s and, one dependent variable $\ln\big(D(BM)\big)$ can be written as:

$$\ln\big(D(BM)\big)\sim\beta_{0,BM}+\beta_{1,BM}\ln\left(\left[a_{blood}(t_s)\cdot\theta_{BM,BM}+\frac{S\cdot C_{RM}(t_s)}{V_{RM}}\cdot\theta_{BM,RM}\right]\right)+\beta_{2,BM}\;t_s \qquad \text{(Eq. A15)}$$

with $\beta_{0,BM}$, $\beta_{1,BM}$ and $\beta_{2,BM}$ the regression coefficients.

Once the models are trained and the regression coefficients determined (Table 2), the predicted absorbed doses by the organs or tumor r_k and by the bone marrow can be calculated using the equations A16 and A17, respectively:

$$D(r_k)\sim\exp\left(\alpha_{0,k}+\alpha_{1,k}\ln\left(\frac{S\cdot C_k(t_s)}{V_k}\right)+\alpha_{2,k}\;t_s\right)$$

$$\Leftrightarrow D(r_k)\sim\left[\frac{S\cdot C_k(t_s)}{V_k}\right]^{\alpha_{1,k}}\cdot e^{\alpha_{2,k}\,t_s+\alpha_{0,k}} \qquad \text{(Eq. A16)}$$

$$D(BM)\sim\exp\left(\beta_{0,BM}+\beta_{1,BM}\ln\left(\left[a_{blood}(t_s)\cdot\theta_{BM,BM}+\frac{S\cdot C_{RM}(t_s)}{V_{RM}}\cdot\theta_{BM,RM}\right]\right)+\beta_{2,BM}\;t_s\right)$$

$$\Leftrightarrow D(BM)\sim\left[a_{blood}(t_s)\cdot\theta_{BM,BM}+\frac{A_{RM}(t_s)}{m_{RM}}\cdot\theta_{BM,RM}\right]^{\beta_{1,BM}}\cdot e^{\beta_{2,BM}\,t_s+\beta_{0,BM}} \qquad \text{(Eq. A17)}$$
