# Supplementary material for: Simple model for estimation of absorbed dose by organs and tumors after PRRT from a single SPECT/CT study
Source: EJNMMI Phys. 2021 Aug 26;8:63. doi: 10.1186/s40658-021-00409-z (PMC8390741; doi:10.1186/s40658-021-00409-z)
Supplement: Supplementary file 2 — Additional file 2. Number of cases the patient management would have been different between the MLR-based and standard dosimetry protocols for a given combination of (tf, ts) after the first treatment cycle and after the following ones. [file 40658_2021_409_MOESM2_ESM.docx]

**Additional file 2: Table S1.** Number of cases the patient management would have been different between the MLR-based and standard dosimetry protocols for a given combination of (*t*_f_, *t*_s_) after the first treatment cycle and the following ones.

| **First**  **cycle**  **Following**  **cycles** | | **Eq. for *t*_f_** | ***t*_1_** | ***t*_2_** | ***t*_3_** | **(*t*_1_, *t*_2_, *t*_3_)** | | |
| --- | --- | --- | --- | --- | --- | --- | --- | --- |
|  |  | **SPECT/CT**  **at *t*_s_** | ***t*_1_** | ***t*_2_** | ***t*_3_** | ***t*_1_** | ***t*_2_** | ***t*_3_** |
| **Eq. for *t*_f_** | **SPECT/CT**  **at *t*_s_** | ***Number of cases the patient management would have been different between the MLR-based and standard dosimetry protocols*** | | | | | | |
| ***t*_1_** | ***t*_1_** |  | 5/32 | 1/28 | 3/32 | 5/32 | 3/28 | 0/32 |
| ***t*_2_** | ***t*_2_** |  | 3/32 | 2/28 | 3/32 | 3/32 | 3/32 | 2/32 |
| ***t*_3_** | ***t*_3_** |  | 6/32 | 3/28 | 5/32 | 6/32 | 4/28 | 4/32 |
| **(*t*_1_, *t*_2_, *t*_3_)** | ***t*_1_** |  | 6/32 | 1/28 | 4/32 | 6/32 | 4/28 | 3/32 |
|  | ***t*_2_** |  | 3/32 | 2/28 | 2/32 | 3/32 | 3/28 | 3/32 |
|  | ***t*_3_** |  | 4/32 | 2/28 | 5/32 | 4/32 | 3/28 | 5/32 |
